# Microbiome Management by Biological and Chemical Treatments in Maize Is Linked to Plant Health

**DOI:** 10.3390/microorganisms8101506

**Published:** 2020-09-30

**Authors:** Peter Kusstatscher, Wisnu Adi Wicaksono, Dhivya P. Thenappan, Eveline Adam, Henry Müller, Gabriele Berg

**Affiliations:** 1 Institute of Environmental Biotechnology, Graz University of Technology, 8010 Graz, Austria; wisnu.wicaksono@tugraz.at (W.A.W.); eveline.adam@saatzuchtgleisdorf.at (E.A.); henry.mueller@tugraz.at (H.M.); gabriele.berg@tugraz.at (G.B.); 2 Division of Microbiology, ICAR-Indian Agricultural Research Institute, New Delhi 110012, India; dhivyathenappan@gmail.com; 3 Biotenzz GmbH, 8010 Graz, Austria

**Keywords:** *Zea mays*, maize, corn, rhizosphere, 16S rRNA gene, ITS, fungicide, plant growth promoting rhizobacteria

## Abstract

The targeted application of plant growth-promoting rhizobacteria (PGPR) provides the key for a future sustainable agriculture with reduced pesticide application. PGPR interaction with the indigenous microbiota is poorly understood, but essential to develop reliable applications. Therefore, *Stenotrophomonas rhizophila* SPA-P69 was applied as a seed coating and in combination with a fungicide based on the active ingredients fludioxonil, metalaxyl-M, captan and ziram. The plant performances and rhizosphere compositions of treated and non-treated maize plants of two field trials were analyzed. Plant health was significantly increased by treatment; however, overall corn yield was not changed. By applying high-throughput amplicon sequencing of the 16S rRNA and the ITS genes, the bacterial and fungal changes in the rhizosphere due to different treatments were determined. Despite the fact that treatments had a significant impact on the rhizosphere microbiota (9–12%), the field site was identified as the main driver (27–37%). The soil microbiota composition from each site was significantly different, which explains the site-specific effects. In this study we were able to show the first indications how PGPR treatments increase plant health via microbiome shifts in a site-specific manner. This way, first steps towards a detailed understanding of PGPRs and developments of consistently efficient applications in diverse environments are made.

## 1. Introduction

Plants live in close interaction with microorganisms and are therefore recognized as holobionts [1]. This close coevolution developed mainly mutualistic, commensal and neutral, but also pathogenic, interactions [2,3,4]. In particular, beneficial plant–microbe interactions in the rhizosphere were found to be very important throughout the plant’s lifecycle [5]. The rhizosphere harbors high abundances of plant growth-promoting rhizobacteria (PGPR); their modes of action are nutrient uptake, stress protection, induced resistance and plant growth promotion by production of phytohormones [6,7,8]. However, in all agricultural production systems, pathogens originating from soil are a major factor significantly limiting yield and quality in crops. World-wide, farmers are increasingly specialized in the production of distinct crops based on intensive management practice. This has consequences for soil-borne pathogens because they accumulate in soil and enhance disease pressure on crops over time [9]. Fusarium, Rhizoctonia and Verticillium are the most important fungal pathogens in soil, which are difficult to suppress because: (i) they form highly persistent survival structures, (ii) they have a very broad host range, and (iii) specific resistance genes in crops are often not known [10]. Loss in global and local biodiversity is linked to an increase in soil-borne pathogens [11,12,13]. Annually, they cause losses of income of EUR 4 billion and of yield up to 80% in single farms, but the causes of the world-wide spread are not yet fully understood, and effective methods of control are currently lacking [14]. 

Missing crop rotations leads to reduced microbial diversity because of the missing plant-specific food prints in soil; so-called plant–soil feedbacks were identified as one reason [15]. Plant-mediated changes in the soil microbiome can influence the growth of other plants that grow later in the same soil, but they definitively enrich crop-specific pathogens in soil [16]. This demands again more fungicide applications and intense management. A number of synthetic fungicides are applied currently to the field to target soil-borne pathogens, and threaten global biodiversity [13,17]. Therefore, application of microorganisms antagonistic towards soil-borne pathogens are a promising alternative to suppress them and avoid outbreaks and yield losses [18]. Recently, it was shown that pathogens are able to induce disease-suppressive functions in the endophytic root microbiome [19]. Although antagonistic microorganisms against crop pathogens and PGPR were used for decades to improve plant health and increase field yields in agricultural applications, their inconsistent effect in the field is one reason for missing success. The plant-specific composition of the microbiota is one reason for inconsistency [20]. The interaction of PGPR with the indigenous soil microbiota is poorly understood, and is, according to our hypothesis, another reason for that. To understand the interaction between PGPRs and the indigenous microbiota under field conditions, we studied the effect of the interaction of the maize rhizosphere microbiome and the PGPR *Stenotrophomonas rhizophila* SPA-P69 on maize as a model plant [21].

Domesticated forms of *Zea mays* L. (maize or corn) with a total production of 1.1 billion metric tons (2018) is one of the most grown plants for animal feed and human consumption globally. The biggest production areas are America (52.5%), Asia (29.1%) and Europe (11.2%) [22]. Interestingly, the rhizosphere of maize harbors even a cultivar-specific microbiota [23,24]. Soil-borne pathogens, such as *Phythium sp., Rhizoctonia sp.* and *Fusarium sp.*, are a major threat to the plant, especially in the early growing period [13,25]. Therefore, reliable treatments in changing environmental conditions are needed. Treatments based on PGPR previously isolated from the plant rhizosphere could provide the key to such treatments.

The PGPR strain *Stenotrophomonas rhizophila* SPA-P69 (= DSM14405^T^) was previously studied as a plant growth-promoting bacteria in cotton, tomato, oilseed rape and sweet pepper. It was shown to colonize the roots, grow endophytically and, moreover, increase plant health [26]. This PGPR acts as a Stress Protecting Agent (SPA) by producing osmolytes and spermidine [21]. As the treatment field performance of SPA-P69 varied in previous trials and different locations, we decided to investigate this further. Therefore, we conducted field experiments on maize, involving combined treatments of fungicide and SPA-P69, and studied their impact on plant health, growth promotion and plant colonizing microorganisms. In our experiment we investigated the effect of the seed application of *S. rhizophila* SPA-P69 in combination with a fungicide, based on the active ingredients fludioxonil, metalaxyl-M, captan and ziram. Fungicide treatments were previously shown, apart from their effect on plant health, to also influence the rhizosphere and phyllosphere of plants [27]. We evaluated the agronomic parameter (I), strain establishment in the rhizosphere (II), and looked at the global impact of the treatments using different field sites (III). By studying the interactions of biocontrol and PGPR strains with the plant and other control products, valuable information for the reduction of pesticide use can be gained, and first steps for a movement towards more sustainable and integrated agricultural approaches can be made [18].

## 2. Materials and Methods

### 2.1. Seed Treatments and Sampling Strategy

Untreated single hybrid maize (*Zea mays* L.) seeds (cultivar LG3258, Limagrain, Edemissen, Germany) were inoculated with the stress-protecting agent *Stenotrophomonas rhizophila* SPA-P69 (syn. DSM 14405), further mentioned as SPA-P69 in the entire text, obtained from the Strain Collection for Antagonistic Microorganisms (SCAM, Institute of Environmental Biotechnology, Graz University of Technology, Graz, Austria). After cultivation in Nutrient Broth (NBII; Sifin, Berlin, Germany) for 24 h at 30 °C, cells were harvested by centrifuging at 4000× *g* at 4 °C and adjusted to 10^8^ CFU/mL by resuspending in 0.9% NaCl solution. Seeds were submerged in bacterial suspension for four hours at 22 °C under agitation, followed by two washing steps with sterile distilled water and drying for 24 h in a laminar flow cabinet. For the control treatment, bacterial suspension was replaced by a sterile 0.9% NaCl solution. Half of the seeds with or without bacterial coating treatment were additionally coated with a fungicide mix based on the active ingredients fludioxonil, metalaxyl-M, captan and ziram (product names: MAXIM^®^ XL, Syngenta; Korit^®^, Kwizda Agro GmbH), using sacrust as a carrier (Table 1). The used mixture was MAXIM^®^ XL:Captan:Korit in a 1:1:0.6 ratio, and 490 g seeds were coated with a total of 3.5 mL.

Field trials were carried out at two field sites, located in Lower Austria, Austria (Melk, Austria (48°9′20.28″ N; 15°30′45.10″ E) with neutral (pH 6.6–7.2) soil consisting of lime-free loamy sand with average humus content, and in Mitterdorf a.d. Raab, Styria, Austria (47°10′42.29″ N; 15°36′45.16″ E), with a slightly acidic to acidic (pH 4.6–6.5) soil type predominated by lime-free sandy loam with average humus content. The randomized plot design included three replicates per treatment. In late April 220 seeds per plot in Mitterdorf and 180 seeds in Melk were planted in four rows. Four weeks post-planting, emerged plants were counted and the number of plants per row was 40 or 30 individuals (160 or 120 plants per plot/replicate) in Mitterdorf and Melk respectively. The fresh weight of the shoot was assessed from six out of the extracted plants. For microbiome analysis one composite sample per plot (three samples per treatment), consisting of roots with adhering soil from four randomly chosen plants, was collected from both locations. Additionally, four soil samples (10–15 cm depth) were taken from random locations on the field. At the end of October, corn yield was recorded from the two inner rows of a plot after mechanical harvesting.

### 2.2. DNA Extraction and Amplicon Library Construction

After arrival in the lab (cooled and within 24 h), total community DNA was extracted from the samples. Theren, 5 g of the soil and root samples were homogenized using a stomacher (Bagmixer; Intersciences, St. Nom, France) for 3 min using 50 mL of sodium chloride (0.85%). A total of 4 mL of homogenized solution was pelleted and further used for DNA extraction. The total community DNA was extracted using the FastDNA SPIN Kit for Soil and the FastPrep Instrument (MP Biomedicals, Santa Ana, CA, USA) following the manufacturer’s protocol. DNA was quality-checked using a Nanodrop 2000 (Thermo Scientific, Wilmington, DE, USA) and stored at −20 °C for PCR reactions.

Isolated DNA from two locations, four treatments and bulk soil samples was used for amplification of the 16S rRNA gene V4 hypervariable region and ITS1 region. The 515f/806r primer pair (515f: 5′-GTGYCAGCMGCCGCGGTAA-3′; 806r: 5′-GGACTACNVGGGTWTCTAAT-3′) for bacteria and ITS1f/ITS2r (ITS1f: 5′-CTTGGTCATTTAGAGGAAGTAA-3′; ITS2r: 5′-GCTGCGTTCTTCATCGATGC-3′) for fungi were used [28,29,30]. All PCR reactions were performed in triplicates using Taq&Go PCR-Mix (MP Biomedicals, CA, USA), primer pairs, template DNA and water. The PCR mix was amplified in 35 cycles at 94 °C denaturation for 45 s, then underwent 50 °C annealing for 60 s and 72 °C elongation for 90 s. The amplicons were purified using the Wizard SV Gel and PCR Clean-Up System (Promega, Madison, WI, USA) and pooled in equimolar concentrations. The paired-end Illumina MiSeq sequencing (2 × 300 bp) of the barcoded Illumina library was performed by GATC Biotech (Berlin, Germany).

### 2.3. Bioinformatic Analysis

Paired-end reads were quality checked and demultiplexed using cutadapt [31]. Bioinformatic analysis for amplicon sequencing analysis was performed using the open-source QIIME2 v. 2018.4.0 pipeline [32]. Primer sequences were removed using cutadapt [31], forward and reversed reads were joined, and feature tables and representative sequences (amplicon sequences variants (ASVs)) were generated using the DADA2 algorithm using quality filtering, denoising and chimeric filtering steps in QIIME2 [33]. The generated ASVs were classified using the vsearch algorithm, and SILVA v. 132 and UNITE v. 8.0 were used as bacterial and fungal reference databases, respectively [34,35,36,37].

### 2.4. Statistical Analysis

The R version (R Core Team, Vienna, Austria (2017)) was used to perform statistical analysis and create Non-metric Multi-dimensional Scaling (NMDS) plots. Differences in agronomic data were tested using a pairwise *t*-test. The resulting ASV tables and taxonomy from QIIME2 were imported into R via phyloseq [38]. Bacterial and fungal analysis were performed by rarefying the dataset to the lowest amount of sequences in each dataset by randomly selecting subsets of sequences to account for the uneven sequencing depth. Alpha diversity significance (*p* < 0.05) was tested using Kruskal–Wallis tests, followed by pairwise comparisons and Tukey HSD post-hoc test. Beta diversity based on Bray–Curtis dissimilarities was tested using permutational analysis of variance (PERMANOVA, 999 permutations). The distance matrices were visualized by using a non-metric multidimensional scaling (NMDS) plot. Differences in taxa abundance between treatments were determined by DESeq2 analysis [39]. Prior to DESeq2 analysis, ASVs with relative abundance less than 0.1% were removed. The significant differences below 0.05 were *p* value-adjusted using the Benjamini–Hochberg method for multiple testing.

### 2.5. qPCR Analysis of SPA-P69 Gene Copy Numbers

Total DNA extracts from the samples were further used to specifically quantify *S. rhizophila* SPA-P69 by real-time PCR using TaqMan^®^ and the primers Strh_for (5′-CACCTGAAAGAATGTAGGAGTGG-3′) and Strh_rev (5′-CTCGCTCTTTTCCCTAGTGC-3′) in combination with the probe Strh_pr (5′-CAGGGAAGCAAGCGCACCGT-3′). The quantification was performed on a Corbett Research^TM^ thermocycler (Rotor-Gene 6000, Corbett Research, Cambridge, UK). The following approach was used to perform the qPCR (total volume 10 µL): 5 µL TaqMan^®^ Environmental Master Mix 2.0; 0.05 µL of Primers (fwd/rev); 0.2 µL Probe; 0.5 µL Sample; 4.2 µL nuclease-free water. The temperature program for the qPCR was composed of denaturation at 95 °C for 10 min, followed by 45 cycles of annealing at 95 °C for 0.4 min and extension at 60 °C for 1 min. The standard regression curve was obtained using the 160 bp long target fragment with known concentration and further 1:10 dilutions. Three replicates of each standard dilution were prepared to generate a mean value. The standard regression curve was employed to determine the gene copy numbers in the analyzed samples. All PCR reactions were performed in triplicates.

## 3. Results

### 3.1. Plant Growth Performance and SPA-P69 Colonization in the Rhizosphere

**Figure 1:**
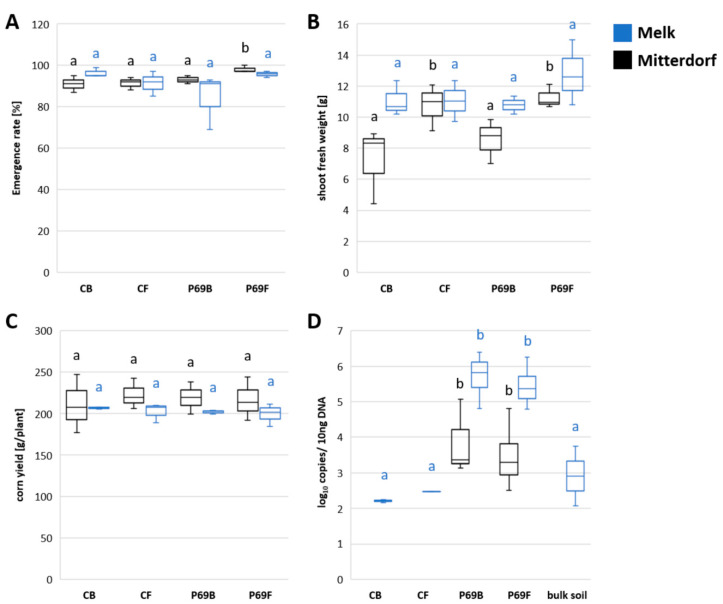
Plant and microbiota data (rating, qPCR). Emergence rate (**A**), shoot fresh weight (**B**), corn yield (**C**) and SPA-P69 copy numbers observed for different maize treatments (**D**). Treatments CB (control), CF (fungicide), P69B (SPA-P69) and P69F (SPA-P69 and fungicide) are labeled on the x-axis. Significance was tested using pairwise t-test within locations (indicated by letters). A detailed statistic report can be found in Table S1.

To investigate the treatment effect on maize plants, different plant parameters were observed during the field trial. While no effect of the treatment on corn yield was observed between treatments, a significantly higher emergence rate and shoot fresh weight was observed for the combined treatment of fungicide and SPA-P69 in Mitterdorf. Moreover, shoot fresh weight was significantly increased by fungicide treatment, especially with a combined treatment using stress-protectant strain SPA-P69 (Figure 1A–C). When copy numbers of SPA-P69 were analyzed for all samples via qPCR, a significantly (*p* < 0.01) higher abundance was found in SPA-treated samples (1000-fold increase). Therefore, the colonization of the rhizosphere by the SPA-P69 strain was successful. Interestingly, while in Melk *Stenotrophomonas sp.* was also detected in control plants, in Mitterdorf only the treated samples showed detectable copies of SPA-P69 (Figure 1D).

### 3.2. Microbial Diversity Analysis Comparing the Two Sites

**Figure 2:**
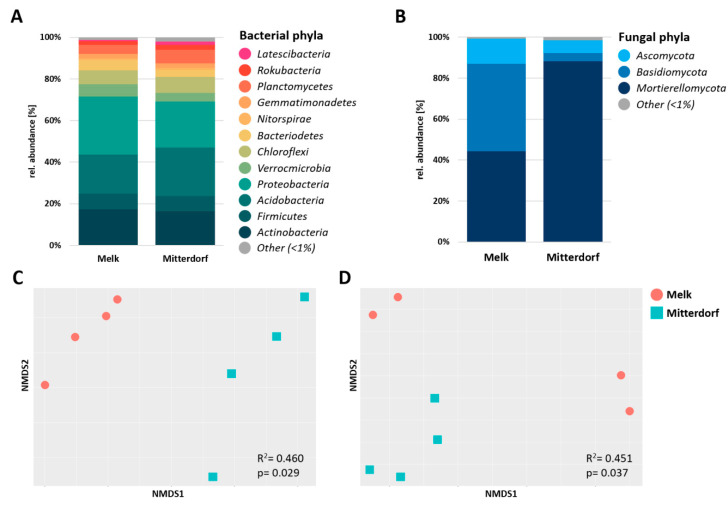
Taxonomic composition (**A,B**) and observed beta diversity (**C,D**) of bulk soil in two different locations. Beta diversity is shown as non-metric multidimensional scaling (NMDS) of Bray–Curtis dissimilarity measures. Significance was tested using PERMANOVA.

Totals of 927,021 and 166,333 high-quality reads were recovered after the filtering and removing of non-target taxa for 16S rRNA and ITS gene amplicon datasets, representing 7012 and 2395 bacterial and fungal ASVs, respectively (Tables S2 and S3). The taxonomic assignment of ASVs indicated that the bulk soil was dominated by six bacterial phyla (>80% of total reads), i.e., Actinobacteria, Firmicutes, Acidobacteria, Proteobacteria, Verrucomicrobia and Chloroflexi (Figure 2A). The relative abundances of Acidobacteria and Chloroflexi were relatively higher at the Mitterdorf site (23.2% and 7.6%, respectively) when compared to the Melk site (18.8% and 6.8%, respectively). In contrast, Proteobacteria and Verrucomicrobia relative abundances were higher in Melk (28% and 5.8%) in comparison to Mitterdorf (22.1% and 4.2%, respectively). In deeper taxonomic resolution, five bacterial classes, namely Alphaproteobacteria, Gammaproteobacteria, Subgroup 6, Actinobacteria and Thermoleophilia, dominated both soils, which accounted 50.7% and 42.7% of the total reads at the Mitterdorf and Melk sites, respectively. The relative abundances of Gammaproteobacteria and Subgroup 6 were higher in Melk (11.9% and 11.8%, respectively) in comparison to the Mitterdorf site (8.7% and 8.6%, respectively). Three fungal phyla, i.e., Mortierellomycota, Basidiomycota and Ascomycota, were prevalent in bulk soils from both sites (Figure 2B). These phyla accounted for up to 98.4% and 99.1% of the total reads from bulk soils sampled from the Mitterdorf and Melk sites. Overall, a higher relative abundance of Mortierellomycota was observed in Mitterdorf (88.2%), while Mortierellomycota and Basidiomycota were similarly abundant in Melk (44.2% and 42.8%, respectively). At the fungal class level, the Mortierellomycotina cls Incertae sedis relative abundance was higher at the Mitterdorf site (88.2%) in comparison to Melk (44.2%), whereas Tremellomycetes showed the opposite pattern (2.9% and 38.4%, respectively).

The bacterial and fungal richness in the bulk soil of both locations was assessed using Shannon diversity metrics (H′). Alpha diversity between the locations did not differ significantly (*p* = 0.205) for bacteria (H′ = 6.0 in Melk and H′ = 6.4 in Mitterdorf, Table S2), however the fungal diversity was significantly (*p* = 0.003) higher in Melk (H′ = 2.7, Table S3) compared to Mitterdorf (H′ = 1.9, Table S3). Looking at the non-metric multidimensional scaling (NMDS) of Bray–Curtis dissimilarity measures for the bulk soil from two different experimental locations, an overall clear separation between both locations was found (Figure 2C,D). The calculated beta diversity showed significant difference in the compositions of both bacterial (*p* = 0.029) and fungal (*p* = 0.037) microbiomes, exhibiting variations of 46.0% and 45.1%, respectively. Overall, the sites used in this study harbored different indigenous microbial communities.

### 3.3. Influence of Seed Treatments on the Alpha and Beta Microbial Diversity

**Figure 3:**
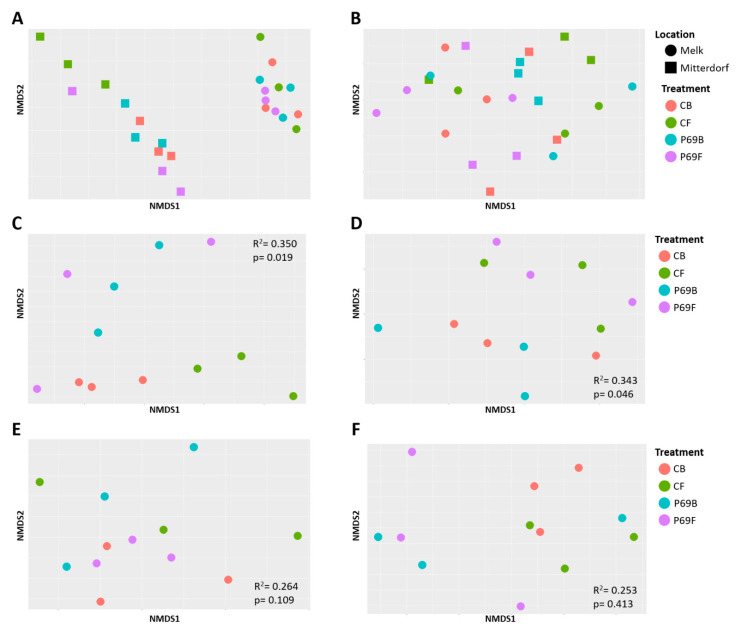
Beta diversity observed for bacterial (**A,C,E**) and fungal (**B,D,F**) communities in treated rhizospheres overall (**A,B**) and for the locations Mitterdorf (**C,D**) and Melk (**E,F**). Beta diversity is shown as non-metric multidimensional scaling (NMDS) of Bray–Curtis dissimilarity measures. Significance was tested using PERMANOVA.

The microbial compositions of rhizospheres from four differently treated maize samples were analyzed. Regarding the beta diversity, field site was found to explain 27.5% of the bacterial variation, while treatment only explained 11.9%. Field site was also the major factor that shaped the fungal community structure by contributing 37.3% of fungal community variation, while seed treatment only explained 8.7% of the variation. Adonis tests indicated the sampling site significantly affected the bacterial and fungal community structure (*p* = 0.001 and *p* = 0.001, respectively). In contrast, seed treatment did not significantly affect the community (*p* = 0.098 and *p* = 0.385, respectively) (Figure 3A,B). Due to the major influence of field site on the community, the rhizospheres of the two locations were further analyzed separately. After separation, the variation within the treatment groups in Melk and Mitterdorf showed clear segregation in NMDS plots (Figure 3C–F).

According to the Shannon index, bacterial richness (α diversity) was slightly affected by treatment in the Mitterdorf site at the 90% confidence interval (*p* = 0.052, Table S4). Post-hoc analysis with Tukey’s tests indicated that the rhizosphere of control plants (CB) had a higher bacterial richness (H′ = 6.1) in comparison to the rhizosphere of fungicide-treated plants at the 90% confidence interval (*p* = 0.063). Moreover, a similar result was observed when the latter was compared to the rhizospheres of SPA-P69- and fungicide-treated plants. In spite of the fact that in Melk the control rhizosphere had a higher bacterial richness (H′ = 5.9, Table S2) in comparison to other treatments (H′ = 5.3–5.8, Table S2), seed treatment did not significantly affect bacterial richness (*p* = 0.767). When beta diversity was assessed by PERMANOVA, treatment significantly affected bacterial community structure (*p* = 0.019) in Mitterdorf, and was shown to explain 35% of the bacterial community variation. Biologically treated and combinatory treated (SPA-P69 and fungicide) samples clustered closer together. Moreover, fungicide-treated samples clustered separately (Figure 3C). Contradicting results were obtained from Melk as beta diversity analysis suggested that seed treatment did not significantly change the bacterial rhizosphere community structure (*p* = 0.109, R^2^ = 0.264) (Figure 3E).

In Mitterdorf, fungal richness was higher in the rhizosphere of control samples (H′ = 2.7, Table S3) in comparison to other rhizosphere samples (H′ = 2.6–2.1, Table S3). A similar trend was also observed in the Melk site. However, ANOVA analysis suggested there was no significant difference in fungal richness between treatments at both of location (*p* = 0.703 and *p* = 0.223, respectively, Table S4). Despite no effect on fungal richness being found, PERMANOVA analysis revealed that seed treatments significantly (*p* = 0.046) affected the fungal rhizosphere community structure in Mitterdorf. Seed treatment was shown to explain 34% of the fungal community variation in the rhizosphere. The rhizospheres of samples treated with fungicide and treated with a combination of fungicide and SPA-P69 tend to cluster together (Figure 3D). In contrast to the result in Mitterdorf, seed treatment did not influence the fungal rhizosphere community structure at the Melk site (*p* = 0.413, R^2^ = 0.213) (Figure 3F). Taken altogether, seed treatment had a site-dependent effect on fungal community structure, as previously described above on the bacterial community structure.

### 3.4. Microbial Composition in Response to Seed Treatments

**Figure 4:**
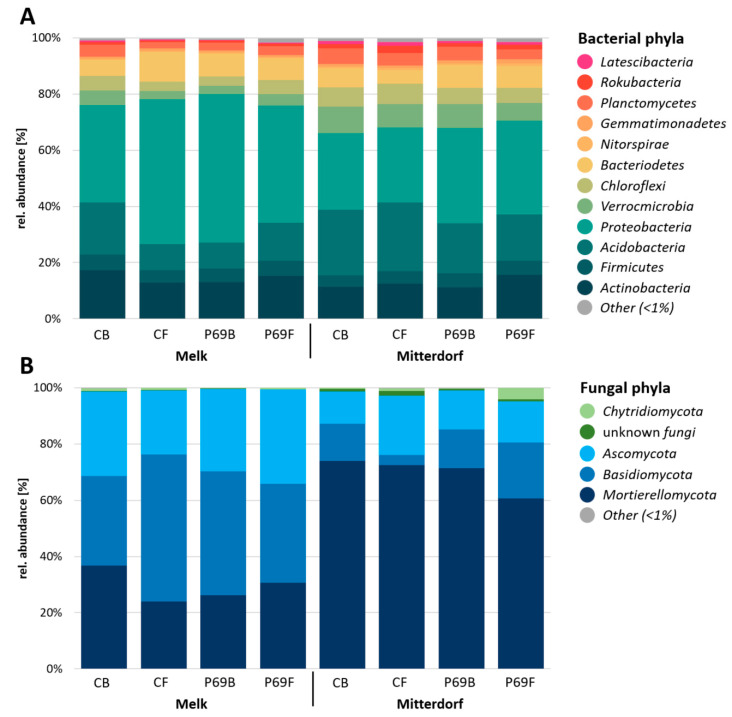
Microbial response in the rhizosphere after seed treatment. Bacterial (**A**) and fungal (**B**) relative taxonomic composition on phylum level is shown for individual treatments.

The taxonomic assignment of ASVs indicated that seven bacterial phyla, including Actinobacteria, Firmicutes, Acidobacteria, Proteobacteria, Verrucomicrobia, Bacteroidetes and Chloroflexi, dominated the rhizosphere regardless of the seed treatment (88.2–94.9% of total reads). When taxonomic profiling was performed at each field site, a distinct bacterial composition was observed between seed treatments (Figure 4A). In Mitterdorf, Proteobacteria abundances were relatively higher in samples treated with PGPR (without and with fungicide, 33.9% and 33.5%) in comparison to untreated and fungicide-treated samples (27.2% and 26.7%). In contrast, Acidobacteria relative abundances displayed the opposite pattern (23.4% and 24.5% in untreated and fungicide-treated samples, respectively; 17.9% and 16.4% in SPA-P69-treated samples and in samples with a combination of PGPR and fungicide, respectively). Different results were observed at the Melk site. Although Proteobacteria abundances were higher in samples treated with SPA-P69 (52.8%) in comparison to the control (34.7%), the abundance in samples treated with fungicide (51.6%) was similar to samples treated with SPA-P69. Moreover, the relative abundances of Acidobacteria in treated samples (9.2–13.4%) were low in comparison to untreated control samples (18.5%).

Fungal taxonomic assignment indicated that three fungal phyla, Mortierellomycota, Basidiomycota and Ascomycota, are dominant in the rhizosphere, regardless of the seed treatment (95.2–99.7% of total reads) (Figure 4B). The relative abundance of Basidiomycota was lower in samples treated with fungicide (3.5%) in comparison to untreated samples (13.1%) in Mitterdorf. In contrast, the opposite result was observed in Melk for Basidiomycota (52.3%—seed treated with fungicide; 31.9%—untreated seed). Moreover, the relative abundances of Mortierellomycota were lower in treated samples (23.9–30.6%) in comparison to untreated samples (36.8%) in Melk. A congruent result was not observed in Mitterdorf, where relative abundances of Mortierellomycota were similar between treatments (71.4–74%), except for samples treated with a combination of fungicide and SPA-P69 (60.7%).

### 3.5. Identification of Responder Taxa in Response to Seed Treatments

Given the observed high impact of the treatments on beta diversities and on the microbial taxonomic composition, DESeq2 analysis was performed to observe in-depth changes triggered by the treatments (Tables S5–S8). The highest number of ASVs differentially abundant were found when comparing samples treated with fungicide and untreated samples in both the field sites (*n* = 19 and *n* = 7 in Mitterdorf and Melk, respectively). Among them, the relative abundances of four (Mitterdorf) and two (Melk) ASVs, belonging to Subgroup 6, were significantly reduced in the rhizospheres of fungicide-treated samples compared to the rhizosphere of the control. In Mitterdorf, the relative abundance of an ASV that belongs to Candidatus Xiphinematobacter (Verrucomicrobiae) consistently decreased after fungicide and combinatory treatment. Additionally, the genus Stenotrophomonas, which the stress-protectant strain SPA-P69 belongs to, was shown to be decreased by fungicide treatment. Moreover, in Melk, the relative abundances of two ASVs that belong to Janthinobacterium and Pedobacter were consistently enriched in the rhizospheres of treated samples compared to the control. In the fungal dataset, generally a low number of significantly increased and decreased taxa were found. One ASV that belongs to Mortierella significantly increased in Mitterdorf after fungicide and combinatory treatment, compared to the control group. Additionally, another ASV from the genus Mortierella significantly increased in Mitterdorf in all treated samples.

## 4. Discussion

Seed treatment using fungicide and/or PGPR improved plant biomass and emergence rates, as well as significantly increasing plant health. Corresponding to these phenotypic effects in plants, we observed statistically significant shifts in the plant-associated bacterial and fungal communities. Another interesting observation is that the field site, mainly the indigenous soil microbiome, was identified as the main driver of the rhizosphere microbiota, as well as the PGPR interaction and effect.

The ability of PGPR to colonize the rhizosphere, the so-called rhizosphere competence, has an effect on plant growth [40]. In this study, seed treatment successfully delivered bacteria to colonize the rhizosphere of maize, as shown using qPCR. However, successful colonization of the PGPR seems to be not the only factor that determines the efficacy of PGPR. We observed no analogous effects on the microbial community composition between the two fields on higher taxonomic levels. Rather, the opposite effect was often observed. This could be linked to the contrasting plant growth effect observed in the field. The mode of action of the PGPRs was intensively studied [8], while their interaction with the indigenous microbiome was neglected. Using PGPR treatments, the microbial community should be changed towards a healthier and more diverse composition [12,41]. In our experiment, the microbial alpha diversity did not significantly increase with different treatments. Nevertheless, treatments were shown to have a high (25–35%) influence on the beta diversities of bacterial and fungal communities in Mitterdorf, where plant growth-promoting effects were observed on the field. The two observed locations differed significantly in their indigenous soil microbiomes. Different indigenous microbial communities are likely due to a complex range of environmental factors, such as the pH, moisture and nutrient availability of each site [42,43,44]. A previous study indicated that SPA-P69 promotes plant growth by modifying fungal communities in the rhizosphere and by eliminating deleterious microorganisms under greenhouse conditions using standard soil [26]. Our study supports these previous findings, as SPA-P69 was able to change rhizosphere microbial communities in Mitterdorf under field conditions. However, no effect was visible in Melk, where also no growth promotion was observed. These findings underline that (i) the composition of the indigenous soil microbiota, which depends also on soil parameters, is crucial for the effect *in planta*, and (ii) inducing a microbiome shift is an important mode of action of PGPR agents.

In comparison to seeds treated with PGPR, fungicide treatment had a higher impact on the microbial community. In alignment with a previous study by Nettes et al. (2016) [45], the significant effect was primarily observed on the fungal community’s structure, but not on the fungal richness. Nevertheless, current studies have also demonstrated the off-target effects of fungicide on soil microbial communities [46,47,48]. In the present study, bacterial richness tends to decrease in response to fungicide treatment. The field trial in Mitterdorf indicated both off-target and site-specific effects of fungicide. Moreover, bacterial community compositions were significantly affected by the fungicide treatment. However, the mechanism by which the fungicide treatment affected the bacterial community’s structure is not clear. Seed treatment with fungicide may eliminate certain bacteria and fungi during the early colonization, then create a new niche or reduce competition for others to colonize [45,49]. Moreover, a higher impact on the bacterial community indicated that bacteria are more responsive to fungicide treatment during early colonization in the rhizosphere. To confirm this, employing multiple sampling timepoints may be useful to investigate the effects of seed treatment on microbial richness.

When the impacts on the microbial community’s composition were assessed in detail, seed treatments were shown to affect various taxa in the rhizosphere, which may be a link to plant growth. A consistent reduction in multiple ASVs belonging to Subgroup 6, from the phylum Acidobacteria, commonly found in soil [50], was observed. A similar effect was found in both locations, however not in a combined treatment with PGPR strain SPA-P69 and fungicide. Moreover, a significant decrease in Stenotrophomonas, the same genus SPA-P69 belongs to, was observed after fungicide treatment. While SPA-P69 treatment alone had no significant effect on plant performance, a combinatory treatment with the fungicide, however, significantly increased the emergence rate and fresh weight, as well as the population density, of Stenotrophomonas. Our data further showed a significant decrease in Candidatus_Xiphinematobacter, from the family Verrucomicrobiae, in seeds treated with fungicide with and without PGRP, which also improved plant biomass. Candidatus_Xiphinematobacter was previously found to be an endosymbiont of plant-parasitic nematodes [51,52]. Moreover, efficient treatments had further significant effects on several Mortierella ASVs. The genus Mortierella is generally believed to be plant-beneficial, and is moreover applied to control plant diseases and root-knot nematodes [53,54,55]. We speculated that seed treatment may also have an indirect impact on plant-parasitic nematodes, which leads to the improvement of plant biomass. This could be an interesting target for further investigations to assess the off-target impact of fungicide treatment on the nematode community, due to shifts in the microbial community.

## 5. Conclusions

Our study highlights how microbial changes in the rhizosphere are linked to plant growth performance and health. Improved plant performance was associated with significant changes in the plant microbial community. We have also demonstrated the off-target effect of pesticides on the bacterial community’s richness and structure, providing evidence of the adverse effects of fungicide use. Using microbial inoculant in combination with fungicide treatments, an improved plant performance can be achieved and linked to induced microbial shifts. Moreover, the challenges of the varying performances of treatments in changed field conditions were shown. Therefore, not only is a field experiment to determine optimum inoculum concentration and timing to achieve optimum performance in the field suggested, but so too is an analysis of the indigenous soil microbiota.

## Figures and Tables

**Table 1:** Overview of treatments used in two field trials. The used fungicide mixture was MAXIM^®^ XL:Captan:Korit in a 1:1:0.6 ratio.

Table	PGPR Strain	Fungicide
CB	No	No
CF	No	YES
P69B	*Stenotrophomonas rhizophila* SPA-P69	No
P69F	*Stenotrophomonas rhizophila* SPA-P69	YES

## Data Availability

The 16S rRNA gene and ITS gene raw reads obtained from the sequencing company were deposited at the European Nucleotide Archive (ENA) under the project number PRJEB39535.

## Supplementary Materials

The following are available online at https://www.mdpi.com/2076-2607/8/10/1506/s1, Table S1: Statistical evaluation of agronomic parameters and qPCR measurements tested using pairwise *t*-Test, Table S2: Sequencing overview of 16S amplicons for each sample and observed alpha diversity, Table S3: Sequencing overview of ITS amplicons for each sample and observed alpha diversity, Table S4: Statistical evaluation of explained impact of different factors on bacterial and fungal diversity, Table S5: DESeq2 analysis of bacterial ASVs on the site Mitterdorf. Significantly different ASVs with log-change above 2 are shown, Table S6: DESeq2 analysis of bacterial ASVs on the site Melk. Significantly different ASVs with log-change above 2 are shown, Table S7: DESeq2 analysis of fungal ASVs on the site Mitterdorf. Significantly different ASVs with log-change above 2 are shown, Table S8: DESeq2 analysis of fungal ASVs on the site Melk. Significantly different ASVs with log-change above 2 are shown.

## References

[1] Vandenkoornhuyse P., Quaiser A., Duhamel M., Van A.L., Dufresne A. (2015). The importance of the microbiome of the plant holobiont. New Phytol..

[2] Cordovez V., Dini-Andreote F., Carrión V.J., Raaijmakers J.M. (2019). Ecology and Evolution of Plant Microbiomes. Annu. Rev. Microbiol..

[3] Lambers H., Mougel C., Jaillard B., Hinsinger P. (2009). Plant-microbe-soil interactions in the rhizosphere: An evolutionary perspective. Plant Soil.

[4] Mendes R., Garbeva P., Raaijmakers J.M. (2013). The rhizosphere microbiome: Significance of plant beneficial, plant pathogenic, and human pathogenic microorganisms. FEMS Microbiol. Rev..

[5] Philippot L., Raaijmakers J.M., Lemanceau P., Van Der Putten W.H. (2013). Going back to the roots: The microbial ecology of the rhizosphere. Nat. Rev. Genet..

[6] Bakker P.A.H.M., Doornbos R.F., Zamioudis C., Berendsen R.L., Pieterse C.M.J. (2013). Induced Systemic Resistance and the Rhizosphere Microbiome. Plant Pathol. J..

[7] Berg G., Rybakova D., Grube M., Köberl M. (2015). The plant microbiome explored: Implications for experimental botany. J. Exp. Bot..

[8] Lugtenberg B., Kamilova F. (2009). Plant-Growth-Promoting Rhizobacteria. Annu. Rev. Microbiol..

[9] Bennett J.A., Klironomos J. (2018). Mechanisms of plant–soil feedback: Interactions among biotic and abiotic drivers. New Phytol..

[10] Mendes R., Kruijt M., De Bruijn I., Dekkers E., Van Der Voort M., Schneider J.H.M., Piceno Y.M., DeSantis T.Z., Andersen G.L., Bakker P.A.H.M. (2011). Deciphering the Rhizosphere Microbiome for Disease-Suppressive Bacteria. Science.

[11] Bardgett R.D., Van Der Putten W.H. (2014). Belowground biodiversity and ecosystem functioning. Nature.

[12] Berg G., Köberl M., Rybakova D., Müller H., Grosch R., Smalla K. (2017). Plant microbial diversity is suggested as the key to future biocontrol and health trends. FEMS Microbiol. Ecol..

[13] Katan J. (2017). Diseases caused by soilborne pathogens: Biology, management and challenges. J. Plant. Pathol..

[14] Oerke E.-C. (2006). Crop losses to pests. J. Agric. Sci..

[15] De Long J.R., Fry E.L., Veen G.F., Kardol P. (2019). Why are plant–soil feedbacks so unpredictable, and what to do about it?. Funct. Ecol..

[16] Kostenko O., Van De Voorde T.F.J., Mulder P.P.J., Van Der Putten W.H., Bezemer T.M. (2012). Legacy effects of aboveground-belowground interactions. Ecol. Lett..

[17] Wightwick A.M., Walters R., Allinson G., Reichman S., Menzies N. (2010). Environmental Risks of Fungicides Used in Horticultural Production Systems. Fungicides.

[18] Berg G. (2009). Plant–microbe interactions promoting plant growth and health: Perspectives for controlled use of microorganisms in agriculture. Appl. Microbiol. Biotechnol..

[19] Carrión V.J., Perez-Jaramillo J., Cordovez V., Tracanna V., De Hollander M., Ruiz-Buck D., Mendes L.W., Van Ijcken W., Gomez-Exposito R., Elsayed S.S. (2019). Pathogen-induced activation of disease-suppressive functions in the endophytic root microbiome. Science.

[20] Rybakova D., Wikström M., Birch-Jensen F., Postma J., Ehlers R., Schmuck M., Kollmann R., Köhl J., Berg G. (2020). Verticillium Wilt in Oilseed Rape—the Microbiome is Crucial for Disease Outbreaks as Well as for Efficient Suppression. Plants.

[21] Alavi P., Starcher M.R., Zachow C., Müller H., Berg G. (2013). Root-microbe systems: The effect and mode of interaction of Stress Protecting Agent (SPA) Stenotrophomonas rhizophila DSM14405T. Front. Plant Sci..

[22] FAO FAOSTAT (2018). Food and Agriculture Organization of the United Nations. http://www.fao.org/faostat/en/.

[23] Peiffer J.A., Spor A., Koren O., Jin Z., Tringe S.G., Dangl J.L., Buckler E.S., E Ley R. (2013). Diversity and heritability of the maize rhizosphere microbiome under field conditions. Proc. Natl. Acad. Sci. USA.

[24] Walters W.A., Jin Z., Youngblut N., Wallace J.G., Sutter J., Zhang W., González-Peña A., Peiffer J., Koren O., Shi Q. (2018). Large-scale replicated field study of maize rhizosphere identifies heritable microbes. Proc. Natl. Acad. Sci. USA.

[25] Raaijmakers J.M., Paulitz T., Steinberg C., Alabouvette C., Moënne-Loccoz Y. (2008). The rhizosphere: A playground and battlefield for soilborne pathogens and beneficial microorganisms. Plant Soil.

[26] Schmidt C.S., Alavi M., Cardinale M., Müller H., Berg G. (2012). Stenotrophomonas rhizophila DSM14405T promotes plant growth probably by altering fungal communities in the rhizosphere. Biol. Fertil. Soils.

[27] Cernava T., Chen X., Krug L., Li H., Yang M., Berg G. (2019). The tea leaf microbiome shows specific responses to chemical pesticides and biocontrol applications. Sci. Total Environ..

[28] Caporaso J.G., Lauber C.L., Walters W.A., Berg-Lyons D., Lozupone C.A., Turnbaugh P.J., Fierer N., Knight R. (2010). Global patterns of 16S rRNA diversity at a depth of millions of sequences per sample. Proc. Natl. Acad. Sci. USA.

[29] Parada A.E., Needham D.M., Fuhrman J.A. (2015). Every base matters: Assessing small subunit rRNA primers for marine microbiomes with mock communities, time series and global field samples. Environ. Microbiol..

[30] White T., Bruns T., Lee S., Taylor J., Innis M.A., Gelfand D.H., Sninsky J.J., White T.J. (1990). Amplification and direct sequencing of fungal ribosomal RNA genes for phylogenetics. PCR Protocols: A Guide to Methods and Applications.

[31] Martin M. (2011). Cutadapt removes adapter sequences from high-throughput sequencing reads. EMBnet. J..

[32] Bolyen E., Rideout J.R., Dillon M.R., Bokulich N.A., Abnet C.C., Al-Ghalith G.A., Alexander H., Alm E.J., Arumugam M., Asnicar F. (2019). Reproducible, interactive, scalable and extensible microbiome data science using QIIME 2. Nat. Biotechnol..

[33] Callahan B.J., McMurdie P.J., Rosen M.J., Han A.W., A Johnson A.J., Holmes S. (2016). DADA2: High-resolution sample inference from Illumina amplicon data. Nat. Methods.

[34] Kõljalg U., Nilsson R.H., Abarenkov K., Tedersoo L., Taylor A.F.S., Bahram M., Bates S.T., Bruns T.D., Bengtsson-Palme J., Callaghan T.M. (2013). Towards a unified paradigm for sequence-based identification of fungi. Mol. Ecol..

[35] Nilsson R.H., Larsson K.-H., Taylor A.F.S., Bengtsson-Palme J., Jeppesen T.S., Schigel D., Kennedy P., Picard K., Glöckner F.O., Tedersoo L. (2019). The UNITE database for molecular identification of fungi: Handling dark taxa and parallel taxonomic classifications. Nucleic Acids Res..

[36] Quast C., Pruesse E., Yilmaz P., Gerken J., Schweer T., Yarza P., Peplies J., Glöckner F.O. (2012). The SILVA ribosomal RNA gene database project: Improved data processing and web-based tools. Nucleic Acids Res..

[37] Rognes T., Flouri T., Nichols B., Quince C., Mahe F. (2016). VSEARCH: A versatile open source tool for metagenomics. PeerJ.

[38] McMurdie P.J., Holmes S. (2013). phyloseq: An R Package for Reproducible Interactive Analysis and Graphics of Microbiome Census Data. PLoS ONE.

[39] Love M.I., Huber W., Anders S. (2014). Moderated estimation of fold change and dispersion for RNA-seq data with DESeq2. Genome Biol..

[40] Lugtenberg B.J., Dekkers L.C. (1999). What makes *Pseudomonas* bacteria rhizosphere competent?. Environ. Microbiol..

[41] Adam E., Groenenboom A.E., Kurm V., Rajewska M., Schmidt R., Tyc O., Weidner S., Berg G., De Boer W., Salles J.F. (2016). Controlling the Microbiome: Microhabitat Adjustments for Successful Biocontrol Strategies in Soil and Human Gut. Front. Microbiol..

[42] Berg G., Smalla K. (2009). Plant species and soil type cooperatively shape the structure and function of microbial communities in the rhizosphere. FEMS Microbiol. Ecol..

[43] Costa R., Götz M., Mrotzek N., Lottmann J., Berg G., Smalla K., Götz M. (2006). Effects of site and plant species on rhizosphere community structure as revealed by molecular analysis of microbial guilds. FEMS Microbiol. Ecol..

[44] Fan K., Weisenhorn P., Gilbert J.A., Chu H. (2018). Wheat rhizosphere harbors a less complex and more stable microbial co-occurrence pattern than bulk soil. Soil Boil. Biochem..

[45] Nettles R., Watkins J., Ricks K., Boyer M., Licht M., Atwood L.W., Peoples M., Smith R.G., Mortensen D.A., Koide R.T. (2016). Influence of pesticide seed treatments on rhizosphere fungal and bacterial communities and leaf fungal endophyte communities in maize and soybean. Appl. Soil Ecol..

[46] Fournier B., Dos Santos S.P., Gustavsen J.A., Imfeld G., Lamy F., Mitchell E.A.D., Mota M., Noll D., Planchamp C., Heger T. (2020). Impact of a synthetic fungicide (fosetyl-Al and propamocarb-hydrochloride) and a biopesticide (Clonostachys rosea) on soil bacterial, fungal, and protist communities. Sci. Total Environ..

[47] Kalia A., Gosal S.K. (2011). Effect of pesticide application on soil microorganisms. Arch. Agron. Soil Sci..

[48] Sułowicz S., Cycoń M., Piotrowska-Seget Z. (2016). Non-target impact of fungicide tetraconazole on microbial communities in soils with different agricultural management. Ecotoxicology.

[49] Vasanthakumari M.M., Shridhar J., Madhura R.J., Nandhitha M., Kasthuri C., Janardhana B., Nataraja K.N., Ravikanth G., Shaanker R.U. (2018). Role of endophytes in early seedling growth of plants: A test using systemic fungicide seed treatment. Plant Physiol. Rep..

[50] Naether A., Foesel B.U., Naegele V., Wüst P.K., Weinert J., Bonkowski M., Alt F., Oelmann Y., Polle A., Lohaus G. (2012). Environmental Factors Affect Acidobacterial Communities below the Subgroup Level in Grassland and Forest Soils. Appl. Environ. Microbiol..

[51] Orlando V., Chitambar J.J., Dong K., Chizhov V.N., Mollov D., Bert W., Subbotin S.A. (2016). Molecular and morphological characterisation of Xiphinema americanum-group species (Nematoda: Dorylaimida) from California, USA, and other regions, and co-evolution of bacteria from the genus Candidatus Xiphinematobacter with nematodes. Nematology.

[52] Vandekerckhove T., Willems A., Gillis M., Coomans A. (2000). Occurrence of novel verrucomicrobial species, endosymbiotic and associated with parthenogenesis in Xiphinema americanum-group species (Nematoda, Longidoridae). Int. J. Syst. Evol. Microbiol..

[53] Al-Shammari T.A., Bahkali A.H., Elgorban A.M., El-Kahky M.T., Al-Sum B.A. (2013). The use of *Trichoderma longibrachiatum* and *Mortierella alpina* against root-knot nematode, *Meloidogyne javanica* on tomato. J. Pure Appl. Microbiol.

[54] Eroshin V., Dedyukhina E. (2002). Effect of lipids from Mortierella hygrophila on plant resistance to phytopathogens. World J. Microbiol. Biotechnol..

[55] Zhang K., Bonito G., Hsu C.-M., Hameed K., Vilgalys R., Liao H.-L. (2020). Mortierella elongata Increases Plant Biomass among Non-Leguminous Crop Species. Agronomy.

