# Supplementary material for: Microbiome Management by Biological and Chemical Treatments in Maize Is Linked to Plant Health
Source: Microorganisms. 2020 Sep 30;8(10):1506. doi: 10.3390/microorganisms8101506 (PMC7599774; doi:10.3390/microorganisms8101506)
Supplement: Supplementary file 1 [file microorganisms-08-01506-s001.pdf]

Table S1: Statistical evaluation of agronomic parameters and qPCR measurements tested using pairwise t-Test.

Test set	Category		CB	CF	P69B
		**CF**	0.493	-	-
	Emergence rate	**P69B**	0.15	0.435	-
		**P69F**	0.355	0.116	0.024
		**CF**	0.168	-	-
**complete**	Fresh weight	**P69B**	0.811	0.269	-
		**P69F**	0.021	0.339	0.043
		**CF**	0.74	-	-
	Yield	**P69B**	0.87	0.87	-
		**P69F**	0.94	0.69	0.82
		**CF**	0.434	-	-
	Emergence rate	**P69B**	0.084	0.282	-
		**P69F**	0.915	0.496	0.099
		**CF**	0.98	-	-
**Melk**	Fresh weight	**P69B**	0.85	0.88	-
		**P69F**	0.24	0.28	0.22
		**CF**	0.55	-	-
	Yield	**P69B**	0.53	0.98	-
		**P69F**	0.34	0.7	0.72
		**CF**	0.889	-	-
	Emergence rate	**P69B**	0.414	0.493	-
		**P69F**	0.017	0.021	0.063
		**CF**	0.031	-	-
**Mitterdorf**	Fresh weight	**P69B**	0.353	0.141	-
		**P69F**	0.017	0.713	0.078
		**CF**	0.58	-	-
	Yield	**P69B**	0.70	0.87	-
		**P69F**	0.78	0.78	0.91
		**Bulk_soil**	**CB**	**CF**	**P69B**
	**CB**	1	-	-	-
**qPCR**	**CF**	1	1	-	-
	**P69B**	0.00013	5.90E-05	2.00E-05	-
	**P69F**	6.30E-05	2.90E-05	1.00E-05	1

Table S2: Sequencing overview of 16S amplicons for each sample and observed alpha diversity.

Sample ID	Sample type	Field location	Treatment	Filtered quality sequences	Shannon diversity index
soil_Mitterdorf1_bacteria	soil	Mitterdorf	none	36260	6.3
soil_Mitterdorf2_bacteria	soil	Mitterdorf	none	33070	6.3
soil_Mitterdorf3_bacteria	soil	Mitterdorf	none	16690	5.9
soil_Mitterdorf4_bacteria	soil	Mitterdorf	none	9193	5.6
soil_Melk1_bacteria	soil	Melk	none	21547	6.2

soil_Melk2_bacteria	soil	Melk	none	12472	6.0
soil_Melk3_bacteria	soil	Melk	none	68602	6.8
soil_Melk4_bacteria	soil	Melk	none	37257	6.5
rhizosphere_Mitterdorf_CB1_bacteria	rhizosphere	Mitterdorf	CB	30527	6.2
rhizosphere_Mitterdorf_CB2_bacteria	rhizosphere	Mitterdorf	CB	53169	6.3
rhizosphere_Mitterdorf_CB3_bacteria	rhizosphere	Mitterdorf	CB	14234	5.9
rhizosphere_Melk_CB1_bacteria	rhizosphere	Melk	CB	30068	6.2
rhizosphere_Melk_CB2_bacteria	rhizosphere	Melk	CB	14246	5.1
rhizosphere_Melk_CB3_bacteria	rhizosphere	Melk	CB	40783	6.0
rhizosphere_Mitterdorf_CF1_bacteria	rhizosphere	Mitterdorf	CF	7766	5.5
rhizosphere_Mitterdorf_CF2_bacteria	rhizosphere	Mitterdorf	CF	2790	4.9
rhizosphere_Mitterdorf_CF3_bacteria	rhizosphere	Mitterdorf	CF	5393	5.2
rhizosphere_Melk_CF1_bacteria	rhizosphere	Melk	CF	40360	5.8
rhizosphere_Melk_CF2_bacteria	rhizosphere	Melk	CF	27217	5.2
rhizosphere_Melk_CF3_bacteria	rhizosphere	Melk	CF	11194	5.1
rhizosphere_Mitterdorf_P69B1_bacteria	rhizosphere	Mitterdorf	P69B	11280	5.6
rhizosphere_Mitterdorf_P69B2_bacteria	rhizosphere	Mitterdorf	P69B	32102	6.0
rhizosphere_Mitterdorf_P69B3_bacteria	rhizosphere	Mitterdorf	P69B	19881	5.7
rhizosphere_Melk_P69B1_bacteria	rhizosphere	Melk	P69B	106544	3.8
rhizosphere_Melk_P69B2_bacteria	rhizosphere	Melk	P69B	13501	5.7
rhizosphere_Melk_P69B3_bacteria	rhizosphere	Melk	P69B	48768	6.0
rhizosphere_Mitterdorf_P69F1_bacteria	rhizosphere	Mitterdorf	P69F	6503	5.5
rhizosphere_Mitterdorf_P69F2_bacteria	rhizosphere	Mitterdorf	P69F	31655	6.3
rhizosphere_Mitterdorf_P69F3_bacteria	rhizosphere	Mitterdorf	P69F	51874	6.6
rhizosphere_Melk_P69F1_bacteria	rhizosphere	Melk	P69F	48190	5.5
rhizosphere_Melk_P69F2_bacteria	rhizosphere	Melk	P69F	20570	5.5
rhizosphere_Melk_P69F3_bacteria	rhizosphere	Melk	P69F	23315	6.0

Table S3: Sequencing overview of ITS amplicons for each sample and observed alpha diversity.

Sample ID	Sample type	Field location	Treatment	Filtered quality sequences	Shannon diversity index
soil_Mitterdorf1_fungi	soil	Mitterdorf	none	5541	1.9
soil_Mitterdorf2_fungi	soil	Mitterdorf	none	1293	1.8
soil_Mitterdorf3_fungi	soil	Mitterdorf	none	4181	2.2
soil_Mitterdorf4_fungi	soil	Mitterdorf	none	1944	1.6
soil_Melk1_fungi	soil	Melk	none	1540	2.4
soil_Melk2_fungi	soil	Melk	none	2825	2.8
soil_Melk3_fungi	soil	Melk	none	15208	2.9
soil_Melk4_fungi	soil	Melk	none	5733	2.9
rhizosphere_Mitterdorf_CB1_fungi	rhizosphere	Mitterdorf	CB	3109	2.7
rhizosphere_Mitterdorf_CB2_fungi	rhizosphere	Mitterdorf	CB	1072	3.0

rhizosphere_Mitterdorf_CB3_fungi	rhizosphere	Mitterdorf	CB	9758	2.4
rhizosphere_Melk_CB1_fungi	rhizosphere	Melk	CB	4016	3.1
rhizosphere_Melk_CB2_fungi	rhizosphere	Melk	CB	1936	3.0
rhizosphere_Melk_CB3_fungi	rhizosphere	Melk	CB	7557	2.7
rhizosphere_Mitterdorf_CF1_fungi	rhizosphere	Mitterdorf	CF	6712	3.2
rhizosphere_Mitterdorf_CF2_fungi	rhizosphere	Mitterdorf	CF	3837	2.3
rhizosphere_Mitterdorf_CF3_fungi	rhizosphere	Mitterdorf	CF	5318	2.4
rhizosphere_Melk_CF1_fungi	rhizosphere	Melk	CF	5581	2.9
rhizosphere_Melk_CF2_fungi	rhizosphere	Melk	CF	2948	1.7
rhizosphere_Melk_CF3_fungi	rhizosphere	Melk	CF	4714	2.5
rhizosphere_Mitterdorf_P69B1_fungi	rhizosphere	Mitterdorf	P69B	13190	2.2
rhizosphere_Mitterdorf_P69B2_fungi	rhizosphere	Mitterdorf	P69B	5307	3.0
rhizosphere_Mitterdorf_P69B3_fungi	rhizosphere	Mitterdorf	P69B	5263	1.4
rhizosphere_Melk_P69B1_fungi	rhizosphere	Melk	P69B	4183	2.4
rhizosphere_Melk_P69B2_fungi	rhizosphere	Melk	P69B	6224	2.5
rhizosphere_Melk_P69B3_fungi	rhizosphere	Melk	P69B	10616	2.4
rhizosphere_Mitterdorf_P69F1_fungi	rhizosphere	Mitterdorf	P69F	7158	2.9
rhizosphere_Mitterdorf_P69F2_fungi	rhizosphere	Mitterdorf	P69F	5744	2.7
rhizosphere_Mitterdorf_P69F3_fungi	rhizosphere	Mitterdorf	P69F	7342	1.7
rhizosphere_Melk_P69F1_fungi	rhizosphere	Melk	P69F	1626	2.9
rhizosphere_Melk_P69F2_fungi	rhizosphere	Melk	P69F	4077	2.5
rhizosphere_Melk_P69F3_fungi	rhizosphere	Melk	P69F	780	3.0

Table S4: Statistical evaluation of explained impact of different factors on bacterial and fungal diversity.

Factor	Bacterial richness analysis		Fungal richness analysis	
	F value	P value	F value	P value
Soil only dataset				
Location	2.019	0.205	23.63	0.003
Rhizosphere Mitterdorf dataset				
Treatment	3.97	0.052	0.483	0.703
Rhizosphere Melk dataset				
Treatment	0.384	0.767	1.812	0.223

Table S5: DESeq2 analysis of bacterial ASVs on the site Mitterdorf. Significantly different ASVs with log-change above 2 are shown.

Comparison	log2FoldChange	p_{adj}	Class	Genus
CB:CF	5.3	0.033	*Holophagae*	*uncultured Subgroup 7*
	6.2	0.045	*Gammaproteobacteria*	*Stenotrophomonas*
	6.2	0.006	*Subgroup_6*	*uncultured Subgroup 6*
	6.2	0.001	*Bacteroidia*	*uncultured env.OPS17*
	6.2	0.048	*Subgroup_6*	*uncultured Subgroup 6*
	6.2	0.048	*Gammaproteobacteria*	*Massilia*

	6.2	0.007	*Gammaproteobacteria*	*Ellin6067*
	6.3	0.008	*Subgroup_6*	*uncultured Subgroup 6*
	6.4	0.045	*Gammaproteobacteria*	*Ellin6067*
	6.4	0.002	*Verrucomicrobiae*	*Candidatus Xiphinematobacter*
	6.6	0.001	*KD4-96*	*uncultured KD4-96*
	6.6	0.030	*Bacilli*	*Bacillus*
	6.8	0.031	*Alphaproteobacteria*	*Nordella*
	7.4	<0.001	*Verrucomicrobiae*	*Candidatus Xiphinematobacter*
	7.4	0.010	*Subgroup_6*	*uncultured Subgroup 6*
	7.4	0.048	*Bacteroidia*	*Flavobacterium*
	7.9	0.030	*Gammaproteobacteria*	*Rhizobacter*
	-9.7	0.045	*Verrucomicrobiae*	*Opitutaceae*
CB:P69B	7.7	0.030	*Anaerolineae*	*uncultured Anaerolineaceae*
	7.7	<0.001	*KD4-96*	*uncultured KD4-96*
CB:P69F	7.7	<0.001	*Verrucomicrobiae*	*Candidatus Xiphinematobacter*

Table S6: DESeq2 analysis of bacterial ASVs on the site Melk. Significantly different ASVs with log-change above 2 are shown.

Comparison	log2FoldChange	p_{adj}	Class	Genus
	-23.0	<0.001	*Gammaproteobacteria*	*Janthinobacterium*
	-22.2	<0.001	*Bacteroidia*	*Pedobacter*
CB:CF	-10.3	0.005	*Gammaproteobacteria*	*Luteibacter*
	7.0	0.001	*Holophagae*	*uncultured Subgroup 7*
	8.0	<0.001	*Subgroup 6*	*uncultured Subgroup 6*
	11.0	0.002	*Gammaproteobacteria*	*Achromobacter*
	-20.6	<0.001	*Bacteroidia*	*Pedobacter*
CB:P69B	-20.4	<0.001	*Gammaproteobacteria*	*Janthinobacterium*
	-11.6	0.002	*Gammaproteobacteria*	*Luteibacter*
	9.7	0.020	*Gammaproteobacteria*	*Massilia*
	-21.0	<0.001	*Gammaproteobacteria*	*Janthinobacterium*
CB:P69F	-19.3	<0.001	*Bacteroidia*	*Pedobacter*
	11.5	0.001	*Gammaproteobacteria*	*Achromobacter*

Table S7: DESeq2 analysis of fungal ASVs on the site Mitterdorf. Significantly different ASVs with log-change above 2 are shown.

Comparison	log2FoldChange	padj	Class	Genus
CB:CF	22.0	<0.001	*Sordariomycetes*	*Unidentified Nectriaceae*
	-20.2	<0.001	*Mortierellomycotina cls Incertae sedis*	*Mortierella*

CB:P69F	-21.2	<0.001	*Mortierellomycotina cls Incertae sedis*	*Mortierella*

Table S8: DESeq2 analysis of fungal ASVs on the site Melk. Significantly different ASVs with log-change above 2 are shown.

Comparison	log2FoldChange	padj	Class	Genus
CB:CF	-20.5	<0.001	*Mortierellomycotina cls Incertae sedis*	*Mortierella*
	-21.3	<0.001	*Sordariomycetes*	*unidentified Nectriaceae*
CB:P69B	-22.6	<0.001	*Mortierellomycotina cls Incertae sedis*	*Mortierella*
	21.9	<0.001	*Spizellomycetes*	*Spizellomyces*
	-22.0	<0.001	*Sordariomycetes*	*unidentified Nectriaceae*
CB:P69F	-24.2	<0.001	*Sordariomycetes*	*unidentified Nectriaceae*
	-20.7	<0.001	*Mortierellomycotina cls Incertae sedis*	*Mortierella*
